# Silicone Shoes for the Treatment of Foot Pad Dermatitis (Bumblefoot) in Pet Chickens—A Retrospective Case Series

**DOI:** 10.3390/ani14172581

**Published:** 2024-09-05

**Authors:** Cornelia Konicek, Michaela Gumpenberger, Johannes Peter Schramel

**Affiliations:** 1 Clinical Unit of Internal Medicine Small Animal, Service for Avian and Reptiles, University of Veterinary Medicine Vienna, Veterinärplatz 1, 1210 Vienna, Austria; 2 Clinical Unit of Diagnostic Imaging, University of Veterinary Medicine Vienna, Veterinärplatz 1, 1210 Vienna, Austria; michaela.gumpenberger@vetmeduni.ac.at; 3 Clinical Unit of Anaesthesiology and Perioperative Intensive-Care Medicine, University of Veterinary Medicine Vienna, Veterinärplatz 1, 1210 Vienna, Austria; johannes.schramel@vetmeduni.ac.at

**Keywords:** backyard, poultry, silicone shoes, foot pad dermatitis, pododermatitis, pressure-relieving bandages, avian

## Abstract

**Simple Summary:**

Pet chickens are regularly seeking advanced veterinary care for various clinical concerns. One regular condition is foot pad dermatitis, commonly known as bumblefoot, a painful disease often caused by poor living environments and being overweight. Traditional treatments, like bandages, can be hard to use on outdoor chickens because they become dirty and wet. This study aimed to find a new solution: custom-fit silicone shoes made from 3D-printed molds. Using computed tomography scans, we optimized the shape of silicone shoes that could fit and protect chicken feet quite well. Over three years, these shoes were used on 16 clinical cases of chickens who presented with foot pad dermatitis (FPD). Unlike bandages, these shoes allowed the chickens to stay outside and made it easier to treat their foot lesions without needing daily changes. Most chickens wore the shoes for about 14 days, and owners reported positive experiences. The shoes provided better ventilation, could be re-used, and consistently relieved pressure on the chickens’ feet. Despite some issues, like secondary pressure marks and managing feather regrowth, the silicone shoes were a significant improvement over traditional bandages. In conclusion, silicone shoes are a promising way to help backyard chickens with foot pad dermatitis heal faster and are more comfortable, making their care much easier.

**Abstract:**

Backyard chickens often suffer from foot pad dermatitis (FPD), a condition exacerbated by poor husbandry, nutritional deficiencies, and obesity. Pressure-relieving bandages, commonly used in the treatment of FPD, are impractical for outdoor chickens as they quickly become wet and dirty, necessitating daily changes that are often unfeasible. This retrospective study explores the use of custom-fit silicone shoes created via 3D-printed molds as an alternative to traditional bandages. CT scans were used to design shoes adapted from a design used for birds of prey. Over three years, 16 chickens with varying degrees of FPD were treated. The results demonstrated that silicone shoes were an effective treatment for FPD, allowing outdoor activity and facilitating daily lesion care without frequent bandage changes. The median shoe-wearing period was 14 days, and most owners provided positive feedback. Despite some attachment issues, the occurrence of secondary pressure marks, and feather regrowth challenges, the silicone shoes offered better ventilation, reusability, and consistent pressure relief compared to traditional bandages. This study concludes that silicone shoes are a viable solution for managing FPD in backyard chickens, promoting faster healing and improving owner compliance.

## 1. Introduction

Backyard chickens are regularly presented in specialized avian medicine as well as in small animal practices [1]. Owners seek care for their individual chickens due to various complaints, as backyard chickens are prone to a wide range of diseases. In addition to the risk of diseases, husbandry-related issues such as obesity, nutritional deficiencies, lack of exercise, poor litter quality and hygiene, and inadequate perch design can also occur. These factors favor the development of pathologic conditions of their feet, such as foot pad dermatitis (FPD), a well-known issue in the poultry industry, especially in broiler flocks. Within poultry production, the prevalence of FPD varies widely [2]; on average, 5–10% are affected with severe lesions [3]. The prevalence of this condition in backyard chickens is generally unknown. The risk factors seem to align with those described in poultry farming and other bird species, indicating a multifactorial problem that includes wet and caked litter, nutritional deficiencies, and compromised intestinal health [4], as well as obesity and lack of exercise [5].

Treatment and prevention strategies in the poultry industry primarily focus on husbandry and environmental management measures [6] rather than treating individual birds. However, the need for medical intervention for individual chickens is typically seen in backyard poultry care. The treatment protocols of birds of prey, where foot pad dermatitis is a well-recognized and frequently treated condition [5], are tailored for use in pet chickens. These protocols usually involve a multidimensional approach, utilizing systemic and local medications, pressure-relieving bandages, and husbandry measures [7]. Therapy can be intensive and lengthy, requiring good owner compliance. In addition to medical and surgical interventions, selecting an appropriate perching surface and bandage combination is always recommended. The use of custom-made silicone shoes, attached by bandages, has proven to be superior among the various options available [5]. However, challenges arise in bandage application, particularly in outdoor and moist conditions, with owners often struggling to change bandages or keep chickens indoors. Promising outcomes have been observed with the use of pressure-relieving silicone shoes, attachable without the need for classical bandages, in birds of prey [7]. While the use of a 3D-printed mold to create silicone shoes holds potential benefits over traditional bandages, its application in chickens has not yet been described. However, using such pressure-relieving silicone shoes to treat foot pad dermatitis in backyard chickens could be a significant innovation. These shoes could provide a marked improvement from a therapeutic and ethical standpoint as they do not absorb water, enhance ventilation, retain their shape and height, are washable, and allow for local treatment without needing to remove them, thereby reducing the need for frequent bandage changes. Our hypothesis posited that silicone shoes, customized for chicken feet using a 3D-printed mold and attachable without traditional bandages, offer advantages for both chickens suffering from foot pad dermatitis lesions and their owners.

Therefore, this study aims to provide initial insights into the design and use of silicone shoes in backyard chickens.

## 2. Materials and Methods

Using CT scans, a model and a 3D-printed mold for silicone shoes for chicken feet were designed. The CT scans provided high-resolution images that were critical for capturing the intricate details of the chicken’s foot anatomy. The initial design concept was adapted from the one presented by Rasidi et al. [7] for birds of prey at the ExoticsCon 2020 virtual conference. The dimensions of a laying hen’s feet and the angles of the toes were considered when creating the model. A mold was then used to create silicone-cast shoes, with dimensions and design adjusted iteratively during the study period to accommodate the anatomical particularities of chicken feet. Of the selected designs used for clinical cases, three different sizes were created: small, medium, and large. Whenever necessary, the size was particularly adapted to fit the dimensions of the feet and toes and to improve fixation. The proposed mold design, including the 3D printing data and recommended materials, is provided in the Supplementary Materials. The silicone used for casting the shoes was prepared according to the manufacturer’s instructions. To ensure the proper distribution of silicone within the mold, a vacuum chamber was used for casting, or the small nobs within the mold were filled individually using a syringe and needle. The shore A hardness for the silicone was 20 ± 2, its tear resistance was 2.2 N/mm^2^, elongation at break 420%, processing time 6–8 min at room temperature, and curing time approximately 60 min at room temperature.

For these retrospective analyses, medical records from pet chickens with FPD that were treated with silicone shoes instead of traditional bandages between October 2020 and October 2023 were used. These included cases that were either presented due to FPD or with FPD lesions as incidental findings. Criteria for using silicone shoes included chickens with FPD lesions graded from I to V, according to Doneley et al. [8], in addition to good owner compliance.

This is a retrospective study using medical records of the respective chickens to collect relevant information, including admission cause, breed, age, gender, body weight and score, medical history, FPD score and lesion description, treatment regime, additional findings, outcome, owner experiences, and any problems or difficulties encountered in mounting and wearing the silicone shoes. To evaluate the outcomes in each case, we recorded whether and when the FPD lesions completely healed, noted any recurrence of the condition, and assessed for any long-term issues with the feet after the lesions had healed. Long-term issues included a lack of grasping ability due to tendon rupture or stiffness of the phalangeal joints caused by changes in bone structure or osteoarthrosis.

Descriptive statistics (mean and median value of the wearing period of the silicone shoes until the FPD lesions healed) were computed using Microsoft Excel 365 (Version 2402).

## 3. Clinical Cases

### 3.1. Animals

Between October 2020 and October 2023, comprehensive medical records of 16 chickens were available, all of which were provided with the respective, at that time, most recent silicone shoe design. Throughout the observed period, the shoe design underwent a total of 15 adaptations in theory, with three designs selected for clinical use. These changes were based on clinical experience, emerging issues, treatment progress, and feedback from both chickens and owners.

The silicone shoes were used on a total of 14 hens and 2 roosters. Among these cases, 12 were presented due to clinical signs associated with FPD, while FPD was an incidental finding in four cases. The majority (9/16) had lesions affecting only one side, whereas seven chickens had lesions on both feet. The grading of FPD, according to Doneley et al. [8], revealed four cases with a score of 2, eight cases with a score of 3, three cases with a score of 4, and one case with a score of 5 (Table 1).

A primary cause was noted when the chicken was presented due to lameness or swelling of the feet. Incidental findings were recorded when chickens were presented for other reasons, and FPD lesions were found, in addition, without respective clinical signs.

The most affected chickens were heavy individuals belonging to heavy breeds. The median body weight of the chickens was 3047 g, with a range of 1484–4340 g. The known breeds and range of normal body weight of hens and roosters of each breed [9] are provided in Table 2.

In 10 cases, the owners had more than 1 case of FPD in their flock, while in 6 cases, the respective case was the only one observed. Radiographs of the feet were taken in 5 cases, with pathological findings of the skeletal system present in only 2 cases. One chicken exhibited arthrosis of the proximal joint of the third phalanx, while another had signs of osteomyelitis and multiple arthritic changes in all toes of the proximal phalangeal joint.

The microbiological culture of the FPD lesion was conducted in only one case due to non-improvement after the initial therapeutic intervention. In this case, *E. coli* and anaerobic bacteria (without further classification) were identified.

### 3.2. Treatment

In addition to using silicone shoes for treatment, other therapeutic interventions were selected based on the severity of the case and owner compliance. Local treatments, including foot baths initially using iodine followed by Mallow (*Malva sylvestris*) Flower Tea, were applied in all cases. Ointments were chosen according to the wound characteristics, with dry crusty efflorescence treated with Vitawund^®^ (Haleon-Gebro Consumer Health GmbH 6391, Fieberbrunn, Austria), and open or infected wounds treated with Betadine ointment or Manuka honey. Applying a drainage [10] was necessary in only one case, which was used to flush the infected tissue with gentamicin (10 mg/mL) for several days due to the severity of the infection.

Fifteen chickens received NSAIDs (Meloxicam 1 mg/kg PO BID), while six chickens received systemic antibiotic treatment. In two cases, regional limb perfusion with amoxicillin/clavulanic acid (150 mg/kg), as described by Huckins et al. [11], was performed in addition to systemic antibiotic treatment to control severe infection.

Surgical intervention was required in seven cases, with two cases requiring a second surgery. All surgeries involved the removal of necrotic and purulent debris, with primary closure in four cases and leaving the wound open to heal by secondary intention in three cases due to the extent and severity of the lesions. Seven chickens were hospitalized and initially treated in clinics due to their FPD lesions. All these cases were diagnosed with lesions of at least stage III. Additionally, two cases with stage II lesions were hospitalized and initially treated in clinics, although the decision for hospitalization was based on other factors, specifically upper respiratory tract infections. Nonetheless, all cases necessitated continued treatment for FPD at home.

### 3.3. Use of Silicone Shoes

The initial mounting and customization of the silicone shoes were carried out in clinics for all chickens. They quickly acclimated to their shoes, though some initially showed impaired walking and picking at the supplies, which resolved after a short time, usually within one day. Most owners (14/16) accepted the footwear for their chickens and provided positive feedback. Two owners were dissatisfied with the shoe application. One owner removed the shoes after one day and rejected traditional doughnut bandages as well, while the other owner preferred bandages by the end of treatment.

The minimum wearing period of silicone shoes was, therefore, one day, with a maximum of 35 days, an average of 15.4 days, and a median of 14.0 days. The average duration of shoe wear increased from 12.3 to 28.7 days, corresponding to the severity of the lesions. The entire treatment regimen is summarized in Table 3 in accordance with the assigned score.

**Figure 1:**
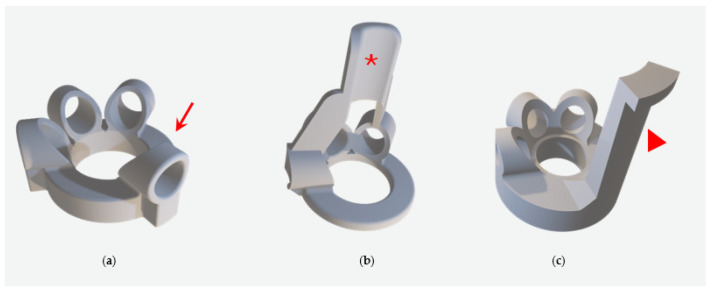
(**a**) The first design created, as described in “birds of prey” by Rasidi 2020, proved problematic. Due to the shorter length of the first toe and its steeper angle in chickens compared to raptor species, the toe slipped out (red arrow), and the shoes did not stay in place. Two different versions were created (**b**,**c**). Instead of the slipper for the 1st toe, a silicone flap attached to the cranial aspect ((**b**), red asterisk) and caudal aspect ((**c**), red arrowhead) of the feet was created to help secure the shoe on the chicken’s feet. However, this design proved impractical due to its rigidity during motion.

**Figure 2:**
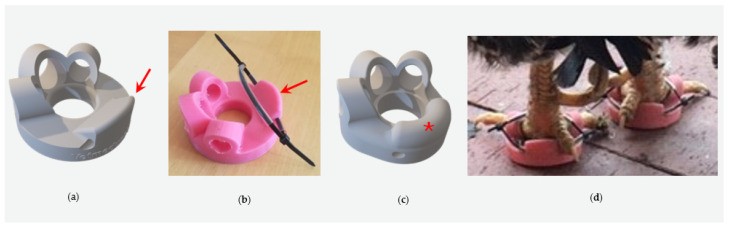
A third version was created (**a**,**b**). A wedge was designed with a gap (red arrow) to accommodate a plastic tube and a cable tie for securing the shoes on the chicken’s feet. However, the fitting was compromised due to the steep edge where the first toe was positioned. Therefore, a fourth version (**c**,**d**) was designed. Instead of the steep edge, a bulge (red asterisk) was created to improve fitting. This design was the first that was clinically used on the depicted rooster (**d**). However, the design still proved to be unstable, and the shoe was occasionally lost.

**Figure 3:**
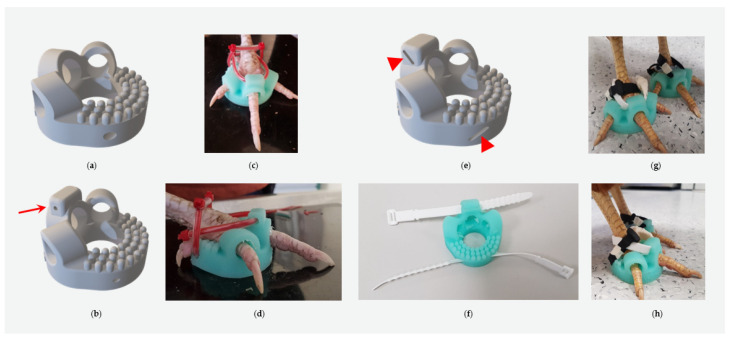
For version five (**a**), instead of the bulge, a nubbin surface on the caudal aspect was created to improve grip and provide a massaging function. However, some chickens still tended to loosen their shoes on the front (2nd to 4th toe). Version six (**b**–**d**), to prevent slipping outside with cranial-pointing toes, had an additional gap (red arrow) created for plastic tube and cable tie attachment on the craniodorsal aspect of the shoe. In the most recent version of the silicone shoes (**e**–**h**), instead of the round gap, a slit (arrowheads) was created to incorporate reusable soft silicone cable ties instead of plastic tubes and normal cable ties.

Difficulties encountered with the silicone shoes included the poor/suboptimal attachment of the shoes due to the short first toe of the chickens and the different angles of the first toe compared to birds of prey. Different attachment types were tried. These included silicone loops, as shown by Rasidi et al. [7], cable ties softened and secured by plastic tubes, Velcro fasters, and finally, adjustable silicone ties were selected (Figure 1, Figure 2 and Figure 3).

Regardless of the versions used, daily monitoring was necessary. Problems arising during the use included shoe loss (8/16), pressure marks from ties (4/16), and painful feather regrowth in feathered leg breeds (2/16).

Compared to doughnut bandages, the advantages of silicone shoes included no required changes, the ability to administer local treatment with the shoes on, allowing all chickens to walk outside even in moist weather conditions, washability and, therefore, also reusability, with consistent height and softness provided by the silicone material and absence of moisture accumulation. Most owners reported that their chickens were still able to perch, and two owners specifically noticed subjectively a faster recovery and improved walking with the shoes.

### 3.4. Outcomes

In all cases, the FPD lesions resolved. However, in one case, long-term issues persisted due to toe arthritic changes and loss of tendon function. In two cases, owners reported recurrent conditions, which they were able to manage themselves with ointments, foot care, and occasionally with the use of shoes or bandages as needed.

## 4. Discussion

The use of a 3D-printed mold to create silicone shoes was presented by the veterinary team of Jurong Bird Park and the Keio-NUS Cute Center at the National University of Singapore (NUS) for the use of various species of birds of prey with good acceptance and outcome. The present study describes the application and necessary adaptations to use such 3D-printed molds for silicone shoes on pet chickens.

FPD, commonly known as bumblefoot, is a well-documented issue in poultry production. It frequently occurs in various species of birds under human care. Information on FPD in backyard poultry is limited, and the prevalence among pet chickens is currently unknown. However, some authors suggest that bumblefoot is one of the more common causes of lameness in pet chickens [12]. From cases seen in former years at the clinic, a prevalence of approximately 4% was calculated. The causes of FPD are multifactorial, with many factors applying to backyard poultry as well. Risk factors such as over-conditioning or obesity, combined with the sedentary lifestyle of captive birds, appear to play a particularly significant role in pet chickens. Additionally, heavy-bodied species are known to be at greater risk [13], as observed in the chickens presented in this study, where many heavier-bodied chickens exhibited FPD lesions, with the majority falling into the upper-weight class of their breed. This appears to be commonly associated with husbandry issues, as at least 10 of the 16 cases involved individuals who were not the only ones affected in their respective flocks.

Among the husbandry conditions described by the owners, no single underlying cause was identified that could be related to all cases, despite most chickens in the flock being overweight. This suggests that multiple factors contribute to the development of FPD, and an increased body condition score and weight seem to be a major factor in backyard poultry. Addressing all possible risk factors is crucial in prevention and management, as already described for the poultry industry [14].

FPD generally follows a chronic course with acute complications like active bleeding and inflammation [7]. Various scoring systems exist to describe and stage these lesions, typically assessing macroscopic changes, but further diagnostics like radiography may be necessary for accurate assessment [8]. Due to variations and potential discrepancies in scoring, inter- and intra-individual agreement on scores is often poor [15]. Therefore, scores in this study should be interpreted with caution, as not all chickens underwent radiography due to financial and clinical relevance constraints.

Among chickens that presented because of foot conditions, only one had a mild stage (score 2) of the disease, compared to incidental findings where only one had a more pronounced stage (score 3). Early stages show mild skin changes without clinical signs, often going unrecognized by the owners, while only advanced stages cause severe lameness and reluctance to stand [7], with some requiring emergency presentations [16]. None of the incidental cases required surgery, indicating that routine checks during clinical exams and the education of pet chicken owners can catch lesions early, allowing for less invasive procedures and a better prognosis.

In poultry production, FPD treatment typically involves husbandry changes and preventive measures [4,17,18]. For pet chickens, individualized treatment plans are necessary. Advanced stages often lead to secondary bacterial infections, commonly caused by *Staphylococcus* sp. and *E. coli* [13]. Although antibiotic sensitivity testing is recommended, broad-spectrum antibiotics are often used for severe lesions [8]. Enrofloxacin and amoxicillin are common choices, though their use may be restricted in laying hens or prohibited in certain countries [12]. In this study, systemic antibiotics were required for treating foot pad dermatitis in 6 out of 16 chickens, all with advanced lesions (stages 3 to 5). Specifically, only two out of eight chickens with a score of three received systemic antibiotics. Again, this emphasizes the importance of routinely examining chicken feet to prevent advanced lesions and, therefore, reduce the necessity to use non-licensed broad-spectrum antibiotics. In addition, treatment with analgesics and anti-inflammatory medications is necessary.

Chronic and advanced cases often require surgical intervention to remove infected material and debris, followed by either primary closure or healing by secondary intention [12,19]. In this study, surgical intervention was necessary in 7 out of 16 cases, with 2 cases requiring two procedures each. Debulking and primary closure were effective in four cases, while three required open treatment for further flushing and healing by secondary intention. One case involved using a drainage tube to flush the infected sites with local antibiotics for several days [10].

Following surgical intervention, it is crucial to relieve pressure from the surgery site [8]. This can be achieved with the use of “doughnut” bandages, which evenly distribute pressure across the foot and help keep the surgical site clean. Various bandage designs have been employed to treat bumblefoot in bird species, including insulation foam, wrapped bandages, and dental silicone material [19]. However, these designs are not standardized and become dirty and wet quickly, especially as chickens typically roam freely outdoors. Furthermore, advanced stages of FPD require long treatment periods, ranging from several weeks to up to a year [7]. Keeping affected chickens indoors and clean for an extended period is often impractical for owners to manage. From previous cases seen at this clinic, applying pressure-relieving bandages, even in less severe stages of FPD, resulted in faster recovery and better overall outcomes (personal observations).

To address the drawbacks of traditional bandages, the idea of using silicone shoes, as presented by Rasidi et al. [7], and adapting them for use in pet chickens was pursued. The design underwent several revisions to fit chicken feet properly. The short first toe and its steep angle required a highly flexible design to prevent slipping and necessitated creating distinct left and right shoes. Instead of incorporating the first toe into the slipper design, it was left free to settle on a doughnut-shaped sole of silicone. Initially, cable ties covered by plastic tubes were used to attach the shoes, but practicality was limited as further adjustments were not possible after closing the cable ties, and pressure marks were observed in some cases. As a final adjustment, slots were included for adjustable, reusable, soft silicone-based cable ties. These proved advantageous as they increased the contact surface and were soft. Velcro fasteners were also attempted but were not considered further due to their susceptibility to wet and dirt outdoors. Thus, the current shoe design with adjustable soft silicone cable tie fixation appears to be the most promising, being easy to attach and remove.

The caudal inner surface of the shoes was designed with a nubbin surface to provide grip and some massaging. Another advantage is that the angle of the first toe does not matter. Hence, the model can be used on either the right or left foot.

The selected sizes of the shoes typically fit well with most of the chickens presented. In special cases where individual dimensions were needed, the mold was adapted accordingly to the chicken’s feet. However, only three different sizes have been necessary so far. Therefore, an individual mold for each case was not required, making these shoes useful for veterinarians without the need for numerous sizes in their practices.

The major advantage of silicone shoes over traditional bandages is their ability to be washed and disinfected using commercially available hand soaps and detergents. Additionally, local treatment of the FPD lesions can be performed without removing the shoes. The chickens’ feet were bathed in a diluted povidone–iodine solution and mallow (*Malva sylvestris*) flower tea without affecting the material. Ointment could also be applied without needing to remove the shoes.

Owner compliance played a crucial role in the use of silicone shoes; therefore, these shoes were only considered in cases where owners were willing to participate in using them as an alternative to traditional doughnut bandages. Even in cases where further treatment beyond wearing the pressure-relieving shoes was unnecessary, owners were instructed to check the feet of their chickens daily. Thus far, all chickens adapted quickly to wearing the shoes, and most owners were satisfied with their practicality and the outcomes.

One disadvantage of silicone shoes is the attachment process, especially with the initial designs, which resulted in pressure marks in some cases. However, owners were resourceful and either padded the attachments themselves or removed the shoes overnight to allow the feet to recover. Material loss or defects in the silicone shoes were not observed in any of the cases, giving them an advantage over conventional bandages, which have to be renewed frequently.

Two chicken breeds included in this investigation had feathered legs and toes, which required the removal of feathers for the treatment and application of the shoes. However, as the feathers regrew, the shoes became painful for the chickens and resulted in pressure marks on the feet. This highlights the importance of special care when using silicone shoes on feathered feet species. Options include fitting the shoes with feathers on the feet and toes or removing the shoes as soon as regrowth begins.

The treatment of FPD is acknowledged to be labor-intensive and can be protracted, lasting for several weeks. In this study, the minimum wearing period of the silicone shoes was set at one day, though this was an instance where the owner declined to continue using the shoes. On average, the shoes were worn for 15.4 days, with a median of 14.0 days and a maximum duration of 35 days. On average, the duration of wearing the shoes decreased in less severe cases but increased with higher scores of FPD. This underscores the benefit of detecting and treating early stages. However, significant information cannot be provided due to the uneven and small number of cases. It was generally anticipated that the wearing period of the shoes would be longer. However, the respective chicken feet appeared to have recovered within that timeframe. Nevertheless, owners were advised to regularly inspect their chicken’s feet over the long term. Only two owners reported recurring conditions. In one instance, the chicken had feathered feet, making it seemingly more susceptible to inflammatory processes. The second case involved an owner who was dissatisfied with managing the footwear and removed the shoes prematurely before complete healing.

Ethical considerations formed the basis for each adjustment made to silicone shoes and attachment devices. The shoes were designed to offer a safe and convenient alternative to the traditional bandages otherwise required. None of the chickens were treated with pressure-relieving shoes without a valid indication. Treatment regimens were adjusted in accordance with the current literature recommendations, with less invasive methods prioritized as the first choice of treatment. Surgical interventions were only considered when conservative approaches were deemed or proven to be insufficient.

The main limitation of this study is its retrospective nature. Also, the chickens used had different stages of FPD lesions, leading to inconsistent treatment regimens, and the shoe design changed over the course of the study. In addition, the small sample size and the lack of a control group are limiting factors. Nevertheless, the main purpose of this report was to evaluate the different attachments and securing options needed for silicone shoes on chicken feet and indicate their practicality in the management of foot pad dermatitis. The study showed promising results, and the use of silicone shoes should be further promoted in future clinical cases. The feasibility of designing silicone shoes for other poultry species kept in backyard free-range systems also needs to be evaluated and tested. Currently, there are no empirical data available. To address the limitations of this case series, further studies involving a larger sample size and extended time are necessary to test the effectiveness and advantages of silicone shoes compared to traditional bandages for chickens with FPD. Additionally, using these shoes in chickens or in other avian species suffering from FPD in zoo settings, where they can be observed by visitors, could serve an educational purpose. This could help raise awareness of this common issue, particularly in commercially raised broiler chickens, and highlight concerns related to animal welfare.

## 5. Conclusions

In conclusion, according to our experience, silicone shoes are a favorable treatment option for backyard chickens with FPD who have committed owners. They are best applied in cases of FPD when wounds do not require daily bandaging. They are particularly suited for treating non-severe cases alongside bathing and ointment. In more severe cases, they can be used before surgery to stabilize the condition and begin local and systemic treatment, and after surgery, once the wound no longer requires covering. The wounds appear to heal faster due to improved ventilation, and chickens can be kept outdoors in any weather conditions without compromise. In chicken breeds with feathered legs and toes, particular care must be taken, especially when plucking the feathers prior to attaching silicone shoes. It is essential to remove the shoes as soon as feathers regrow in these cases to prevent discomfort and pressure marks on the feet.

## Figures and Tables

**Table 1:** This table lists the foot pad dermatitis lesions assigned a score based on Doneley et al. (2019).

Score According to Doneley et al. 2019 [8]	Number of Cases	Main Cause	Incidental	Unilateral	Bilateral
I	0	0	0	0	0
II	4	1	3	1	3
III	8	7	1	4	4
IV	3	3	0	3	0
V	1	1	0	1	0
Total	16	12	4	9	7

**Table 2:** The weight of the cases with foot pad dermatitis, classified by their respective breed, compared to the body weight of the breed, as reported in the literature [9].

Breed	Number of Cases	Range Weight Hens (g)	Range Weight Roosters (g)	Weight of Affected Hens (g)	Weight of Affected Roosters (g)
Sulmtaler	3	2500–3500	3000–4000	3040; 3048; 2807	
Egg layer hybrid	2	n.a	n.a	2837; 2250	
Orpington	2	3000–3500	4000–4500	4050; 3010	
Vorwerk	1	2000–2500	2500–3000	2290	
Deutsches Lachshuhn	1	2500–3250	3000–4000	2802	
Bresse–Gauloise mix	1	2000–2500	2500–3000	2390	
Mixed breed	1	n.a	n.a	1484	
Unknown	5	n.a	n.a	2555; 3080; 2850	4340; 3510

**Table 3:** Selected treatment protocols of the respective cases, classified by the score of the foot pad dermatitis (FPD) lesion. The wearing period of pressure-relieving silicone shoes until recovery of the FPD lesion is given in days, including the mean value in parenthesis.

Score	Number of Cases	Surgery	Systemic AB	i.v. Limb Perfusion	Drainage	Hospitalization	Shoes in Days
I	0	0	0	0	0	0	0
II	4	0	0	0	0	2 ^1^	6–18 (12.3)
III	8	4	2	0	0	4	6 ^2^–30 (17.5)
IV	3	2	3	1	0	2	21–35 (28.7)
V	1	1	1	1	1	1	1 (1) ^2^
Total	16	7	6	2	1	9	

^1^ Hospitalization was selected because of factors other than the FPD lesions. ^2^ Due to failing owner compliance, the owner removed the footwear before the lesions resolved; in one case, this was only after 1 day and in another after 6 days.

## Data Availability

Data are contained within the article and the Supplementary Materials. If more information about the data is needed, this will be available upon request to the authors.

## Supplementary Materials

The following supporting information can be downloaded at https://www.mdpi.com/article/10.3390/ani14172581/s1. Three-dimensional printable files (Creative Commons license) for silicone shoes and detailed instructions with links are given. We highly encourage our colleagues to use these shoes to improve chicken health.
