# Supplementary material for: Silicone Shoes for the Treatment of Foot Pad Dermatitis (Bumblefoot) in Pet Chickens—A Retrospective Case Series
Source: Animals (Basel). 2024 Sep 5;14(17):2581. doi: 10.3390/ani14172581 (PMC11394303; doi:10.3390/ani14172581)
Supplement: Supplementary file 1 [file animals-14-02581-s001.zip › Instructions.pdf]

Chicken foot protector V1.0 JPS

Files

A) Mold.stl FDM standard resolution no support PLA Material 25% infill

B) plugMiddle.stl FDM standard resolution no support PLA Material 100% infill

C) plugsSide.stl (2x needed) FDM standard resolution no support PLA Material 100% infill

D) strip.stl (2x needed) FDM standard resolution TPU

The original files correspond to M size.

Size M: Print scale 100% Toe middle is 14mm (16mm for LD) Toe lateral 12mm
Volume 29ml

Size S : Print scale 78% (Toe middle is 11 mm Toe lateral 9.35 mm

Size L: Scale = 120% Toe middle 16.8 mm Toe lateral 14.4 mm Volume 42ml

To get a proper fit of the plugs adapt scale factor, Use exclusive slicer setting

Silicone for casting:

https://amzn.eu/d/8PJPKTl

Use vacuum chamber for casting. Alternatively you have to fill the nobs with a syringe and needle to get the air bubbles out.

Silicone cable ties:

https://amzn.eu/d/eOeRMcn
